# Association between serum immunoglobulin G4 levels and Graves’ ophthalmopathy: a meta-analysis

**DOI:** 10.3389/fendo.2026.1837951

**Published:** 2026-09-10

**Authors:** Rui Zhang, Yandi Huo, Yunan Guo, Junping Li, Anshi Du

**Affiliations:** 1 Department of Ophthalmology, AIER Eye Hospital Group, Aier Eye Hospital (East of Chengdu), Chengdu, Sichuan, China; 2 Department of Ophthalmology, Chengdu University of Traditional Chinese Medicine, Chengdu, Sichuan, China; 3 Department of Ophthalmology, AIER Eye Hospital Group, Chengdu Aier Eye Hospital, Chengdu, Sichuan, China

**Keywords:** biomarker, Graves’ ophthalmopathy, immunoglobulin G, meta-analysis, risk factor

## Abstract

**Objective:**

Graves’ ophthalmopathy (GO) is a severe extrathyroidal manifestation of Graves’ disease (GD) lacking simple and specific biomarkers. Immunoglobulin G4 (IgG4) has attracted increasing attention in autoimmune and fibrotic diseases. This study systematically evaluates the association of serum IgG4 levels with GO and clarifies the altered characteristics of serum IgG4 levels among GO individuals, as well as their association with GO risk and activity.

**Methods:**

Embase, Cochrane Library, PubMed, and Web of Science were searched from inception to December 2025. Two researchers independently performed literature screening, data extraction, and quality assessment using the Newcastle-Ottawa Scale. Statistical analysis used Stata 18.0. Effect sizes were standardized mean difference (SMD) for continuous variables and odds ratio (OR) for dichotomous variables, both with 95% confidence intervals (CI). Either random- or fixed-effects models were applied based on heterogeneity (I² statistic). Sensitivity and subgroup analyses explored heterogeneity sources. Publication bias was evaluated via funnel plots and Egger’s test using R.

**Results:**

Ten studies with 1, 221 subjects (patients with GO, those with GD without ophthalmopathy, and healthy controls) were included. Meta-analysis showed significantly elevated serum IgG4 levels in patients with GO, especially those with active GO, compared to healthy controls (SMD = 1.260, 95%CI:0.681-1.839) and GD patients without ophthalmopathy (SMD = 1.018, 95%CI:0.278-1.758). High IgG4 levels were associated with increased GO risk (OR = 2.808, 95%CI:1.150-6.859). Univariable analysis confirmed IgG4 as a potential risk factor for GO (ES = 4.735, 95%CI:1.582-14.171), but this association was not statistically significant in multivariable analysis after adjusting for confounders. Subgroup analyses showed higher serum IgG4 levels in active versus inactive GO (SMD = 0.911, 95%CI:0.363-1.460, p=0.001).

**Conclusion:**

Serum IgG4 levels are elevated in individuals with GO and closely associated with disease activity. High serum IgG4 levels represent a significant factor associated with increased GO risk. These findings suggest IgG4 may be involved in specific immunopathological processes underlying GO and could serve as a potential biomarker for auxiliary diagnosis, activity assessment, and risk identification. However, the influence of IgG4 on GO may be modulated by other factors, requiring further validation of its role as an independent risk factor.

**Systematic review registration:**

https://www.crd.york.ac.uk/PROSPERO/view/CRD420251078723, identifier CRD420251078723.

## 1 Introduction

Graves’ ophthalmopathy (GO), which is also referred to as thyroid-associated ophthalmopathy or thyroid eye disease, is a complex autoimmune orbital disorder. GO represents the most common extrathyroidal manifestation among patients with thyroid diseases, occurring in roughly 25% to 50% of individuals suffering from Graves’ disease (GD) and, less frequently, in a small subset of patients suffering from Hashimoto’s thyroiditis (1–3). The incidence rate of GO among males is 0.54-0.9 cases per 100, 000 cases annually, and among females, it is 2.67-3.3 cases per 100, 000 cases per year (4, 5). For GO, the most frequently observed clinical symptoms include eyelid retraction, proptosis, strabismus, and diplopia. In severe GO cases, vision may be adversely affected by significant exposure keratopathy or compressive optic neuropathy (6–8).

The core pathogenesis of GO is an aberrant autoimmune response initiated by the body against orbital tissues (2, 9). Serum immunoglobulin G4 (IgG4) is one of the 4 subclasses of immunoglobulin G (IgG1, IgG2, IgG3, IgG4) and is typically present in lower concentrations. In the normal physiological state, IgG4 constitutes roughly 5% of total serum IgG (10). Fluctuations in IgG4 levels are closely linked to the onset and progression of various autoimmune and inflammatory diseases (11–15). IgG4 is generally involved in modulating immune responses within the body and, under certain circumstances, may play a role in suppressing excessive immune reactions (16). Owing to its unique molecular structure and bidirectional immunomodulatory function, in recent years, IgG4 has become one of the core research objects in the fields of medical immunology and clinical disease (10, 17). It has been proposed that IgG4 may be a key subtype that induces hyaluronan synthesis by orbital fibroblasts among individuals suffering from GO (18). Nevertheless, the influence of IgG4 on the onset of GO is not in isolation, possibly acting independently on orbital tissues or synergistically with thyrotropin receptor antibodies (TRAb) to enhance pathological effects (19, 20). Furthermore, an elevated IgG4 level may serve as a potential marker, indirectly reflecting an increase in TRAb production in orbital tissues, thereby participating in regulating the pathological process in GO.

Findings from current studies remain inconsistent. Some studies have indicated that the serum IgG4 level among individuals suffering from GO is pronouncedly elevated (18), and the IgG4 level is positively associated with the activity and severity of GO (21). However, other studies fail to observe such an association (22). Given this inconsistency and ongoing debate, evidence-based medical evidence is currently lacking. Therefore, this study is designed to systematically include peer-reviewed research of high quality to collect reliable evidence concerning the association of serum IgG4 levels with the incidence rate of GO. Accordingly, this study may provide a reference for relevant clinical decisions.

## 2 Methods

### 2.1 Literature search

This study was implemented adhering to the Preferred Reporting Items for Systematic Reviews and Meta-Analyses (PRISMA) statement, and the study protocol was registered on the International Prospective Register of Systematic Reviews (Registration number: CRD420251078723).

Embase, Cochrane Library, PubMed, and Web of Science were thoroughly searched for studies focusing on the association between IgG4 and GO from the commencement of each database up to December 31, 2025. No language restrictions were applied. The following search strategy, which combined MeSH terms with free-text terms, was employed: “Graves Ophthalmopathy” AND “Immunoglobulin G4”. The search strategies for all databases are detailed in **Supplementary Material 1**. All potentially eligible studies were assessed, irrespective of their primary outcome(s) or language of publication. Additionally, the reference lists of key literature published in English were manually examined for relevant studies.

### 2.2 Literature screening and eligibility criteria

The inclusion criteria were as follows: (i) Studies were case-control, cross-sectional, retrospective, or prospective studies; (ii) A case group comprised patients diagnosed with GO, and a control group consisted of patients suffering from GD or healthy individuals; (iii) Studies provided quantitative data on a serum IgG4 level. Studies were excluded if they were: (i) non-original studies; (ii) studies lacking explicit data on a serum IgG4 level; (iii) publications as a preprint or dissertation; or (iv) non-high-quality studies.

Literature was screened in strict accordance with the PRISMA statement, a process employing a three-step procedure to ensure reproducibility and reliability. Initially, two investigators independently searched the specified databases for relevant literature. After the elimination of duplicates, the titles and abstracts of the remaining records were preliminarily assessed to remove studies not meeting the inclusion criteria, with reasons for exclusion documented. Subsequently, the full texts of the remaining potentially eligible studies were assessed by the two researchers independently against the predefined eligibility criteria. All disagreements were reconciled through negotiation or by consulting with a third senior researcher, and reasons for exclusion at this stage were recorded. Finally, the methodological quality of the eligible studies after full-text review. Studies of low quality were removed, and the completeness of the required data was verified. Ultimately, studies incorporated into this analysis were obtained.

### 2.3 Data extraction and quality assessment

Data from the included studies were extracted by two researchers independently utilizing a standardized spreadsheet. Any disagreement was reconciled through discussion or by consulting with a third researcher. The extracted information encompassed the following variables: publication year, surname of first author, country or region, study design, mean age of a case group and a control group, method used for measuring a serum IgG4 level, sample size, and serum IgG4 level (reported in mg/dL). The methodological quality of the included studies was evaluated by two researchers independently utilizing the Newcastle-Ottawa Scale (NOS) (23). This scale is specifically designed to assess the quality of non-randomized observational studies (e.g., case-control studies and cohort studies). The NOS assesses methodological quality across 3 core domains: selection of study population, comparability between groups, and ascertainment of either exposure(s) or outcome(s). Each domain comprises specific items, which are evaluated through a star rating system. A higher total score is indicative of superior methodological quality and a lower risk of bias. Any discrepancy during the assessment process was resolved by jointly re-examining the original literature or via consensus.

### 2.4 Statistical analysis

All statistical analyses were conducted utilizing Stata 18.0. Heterogeneity among the studies included was evaluated utilizing the I² statistic. According to the I² statistic, low, moderate, and high heterogeneity were indicated by an I² value of 25%, 50%, and 75%, respectively (24). When an I² value exceeded 50%, a random-effects model was selected to minimize the impact of heterogeneity. For continuous data, the standardized mean difference (SMD) was employed as a summary statistic, and the SMD alongside its 95% confidence interval (CI) was computed to analyze relevant outcomes. For dichotomous data and risk data, odds ratios (ORs) alongside their 95% CIs were computed for outcome analysis. When heterogeneity was detected, meta-regression, sensitivity analysis, and subgroup analysis would be implemented. The results were displayed in forest plots, and statistical significance was set at *p* < 0.05. Publication bias was evaluated via funnel plots, supplemented by Begg’s test or Egger’s test.

## 3 Results

**Figure 1:**
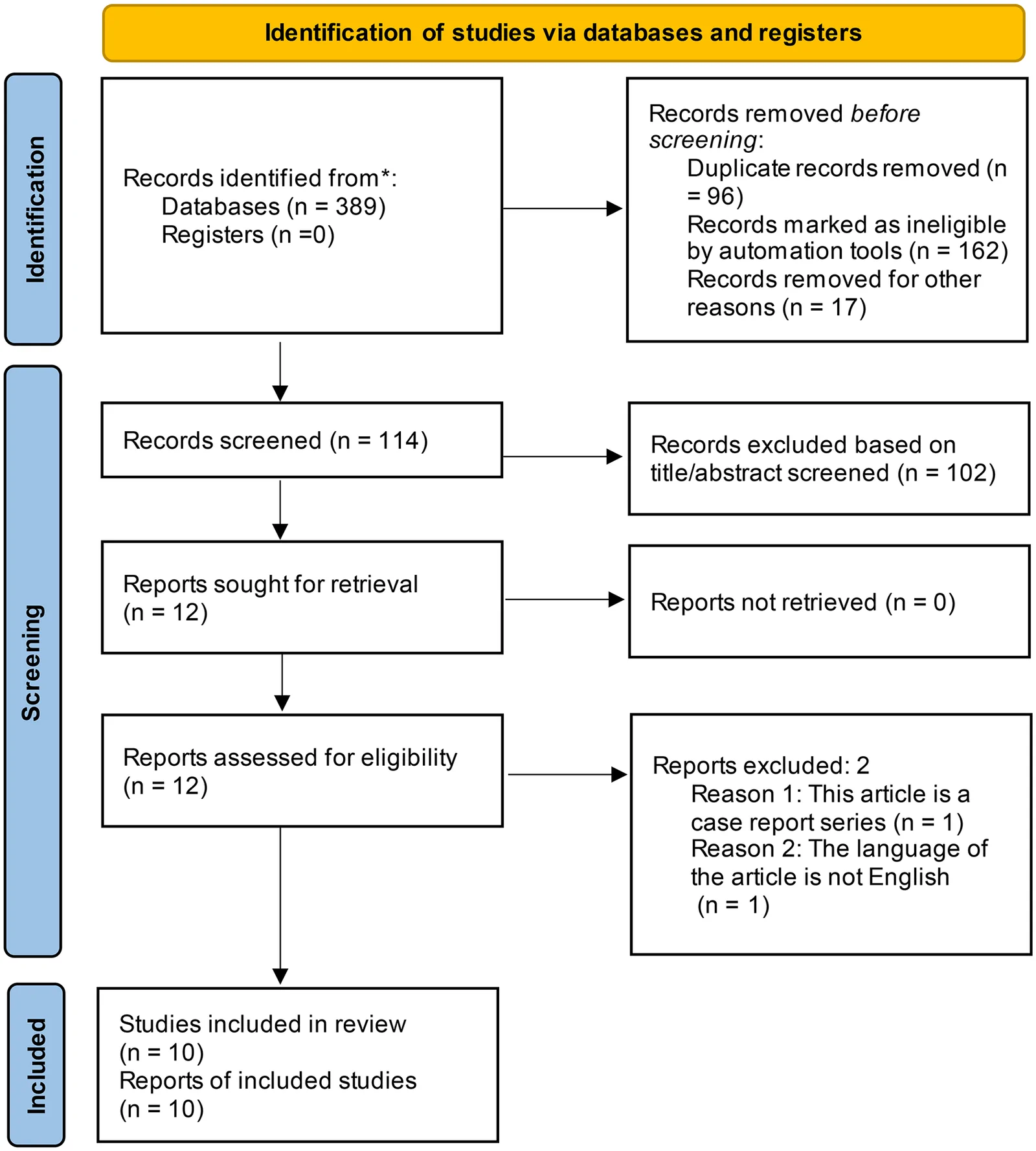
Flow diagram for literature screening.

Initially, a total of 389 records were identified, among which 96 duplicate records were removed. Following the screening of titles and abstracts of the remaining non-duplicate records, 114 articles were considered potentially relevant and identified for full-text review. However, 95 of the articles were subsequently eliminated due to irrelevant abstracts or titles. Consequently, the remaining 19 studies were thoroughly assessed for eligibility. Ultimately, 10 studies (18–22, 25–29) were included. The detailed flow diagram for literature screening is presented in **Figure 1**, and the summarized characteristics of the 10 included studies are provided in **Table 1**.

**Table 1:** Characteristics of included studies.

Author	Publication year	Detection index	Country	Study design	Assay method	GOPatient age (mean ± SD)	GDPatient age (mean ± SD)	Normal patient age (mean ± SD)	Sample size (GO/GD/Normal)	Total sample size
Emre et al	2015	Serum IgG4 levels	Turkey	Cross-sectional study	Turbidimetric assay	41.59 ± 11.33	40.64 ± 10.37	45.04 ± 9.68	32/33/25	90
Carmen et al	2016	Serum IgG4 levels	Romania	Prospective observational study	ELISA kits: Immune enzymatic assay	41.79 ± 14.60	44.26 ± 15.61	NA	29/51	80
Sung et al	2017	Serum IgG4 levels	Korea	Case-control study	Turbidimetric assay	33.2 ± 15.7	39.3 ± 14.2	37.2 ± 14.9	22/42/64	128
Ye et al (27)	2020	Serum IgG4 levels	China	Prospective observational study	Turbidimetric assay	49.38 ± 12.73	NA	NA	185	185
Luo et al	2020	Serum IgG4 levels	China	Cross-sectional study	Turbidimetric assay	47.25 ± 11.14	NA	48.64 ± 11.43	57/50	107
Michal et al	2023	Serum IgG4 levels	Poland	Prospective observational study	ELISA kits: Immune enzymatic assay	54.02 ± 16.02	NA	NA	60	60
Zhao et al (28)	2023	Serum IgG4 levels	China	Retrospective case-control study	Turbidimetric assay	39.89 ± 10.93	NA	40.63 ± 8.12	78/32	110
S. Comi (22)	2024	Serum IgG4 levels	Italy	Retrospective case-control study	Turbidimetric assay	49.2 ± 14.4	41.1 ± 12.9	NA	81/67	148
Michal et al	2024	Serum IgG4 levels	Poland	Cross-sectional study	ELISA kits: Immune enzymatic assay	38.83 ± 11.84	39.96 ± 13.44	NA	12/48/25	85
Deng et al (25)	2025	Serum IgG4 levels	China	NA	Turbidimetric assay	37.0 ± 13.3	33.0 ± 12.6	NA	171/57	228

The 10 included studies were published between 2015 and 2025 and were performed across an assortment of countries in Asia, Europe, and the Middle East, specifically including Turkey, Romania, China, South Korea, Poland, and Italy. The study designs employed comprised cross-sectional, case-control, retrospective, and prospective approaches. The measurement parameter across all studies was serum IgG4 level, quantified using either turbidimetric assays or enzyme-linked immunosorbent assay (ELISA) kits (immune enzymatic assay). The age range of the included patients with GO spanned from 17.5 to 70 years. The total sample sizes of the individual studies varied, with the smallest comprising 60 participants and the largest 228 participants. The methodological quality of the ten included studies was assessed, and each received a score greater than 6 on the NOS, indicating a high quality of methodology.

### 3.1 IgG4 levels in GO compared with GD or normal control groups

**Figure 2:**
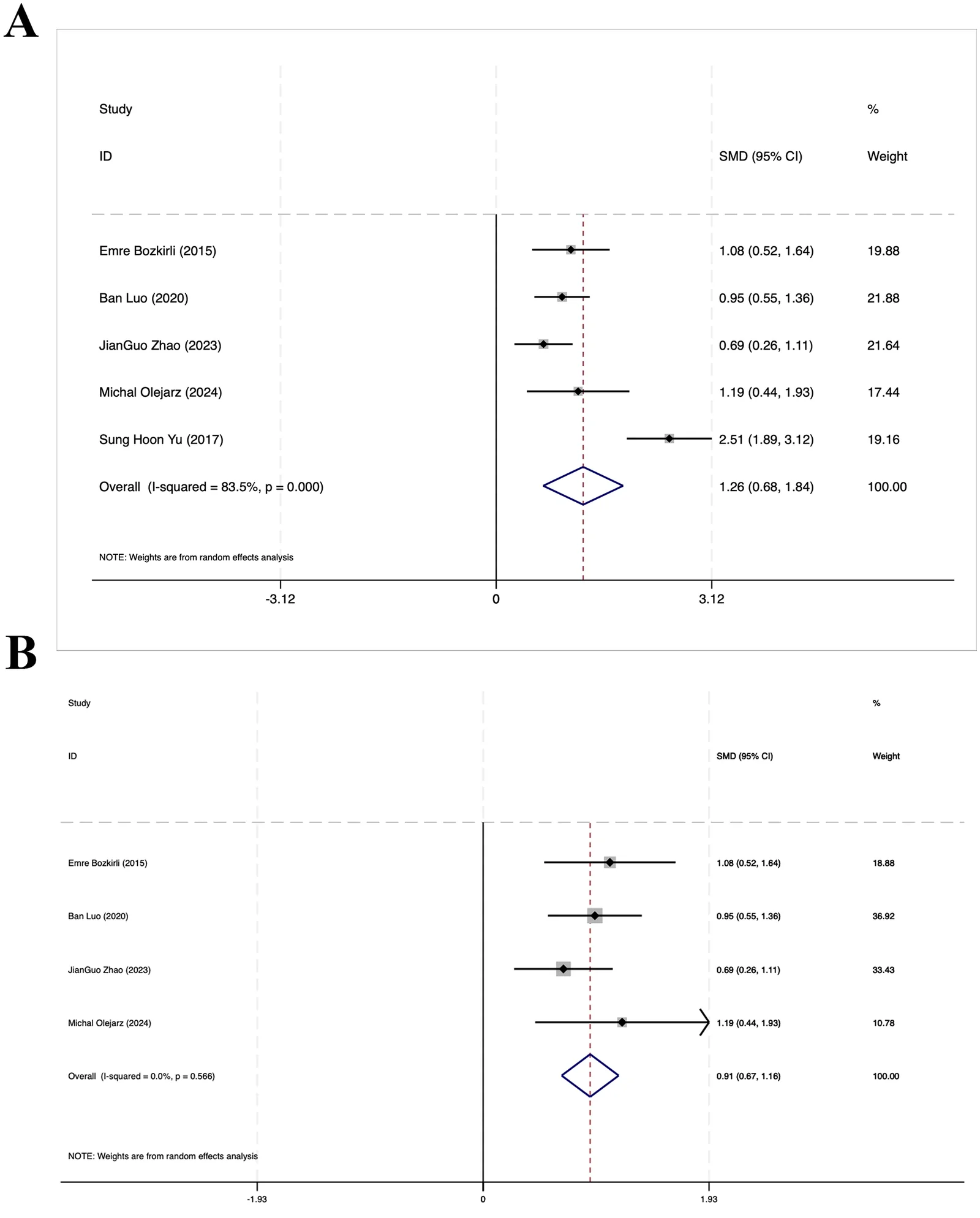
**(A)** Forest plot for the meta-analysis comparing serum IgG4 levels between patients with GO and healthy controls; **(B)** Forest plot for the sensitivity analysis comparing patients with GO and healthy controls after excluding the study by Yu et al. (19).

**Figure 3:**
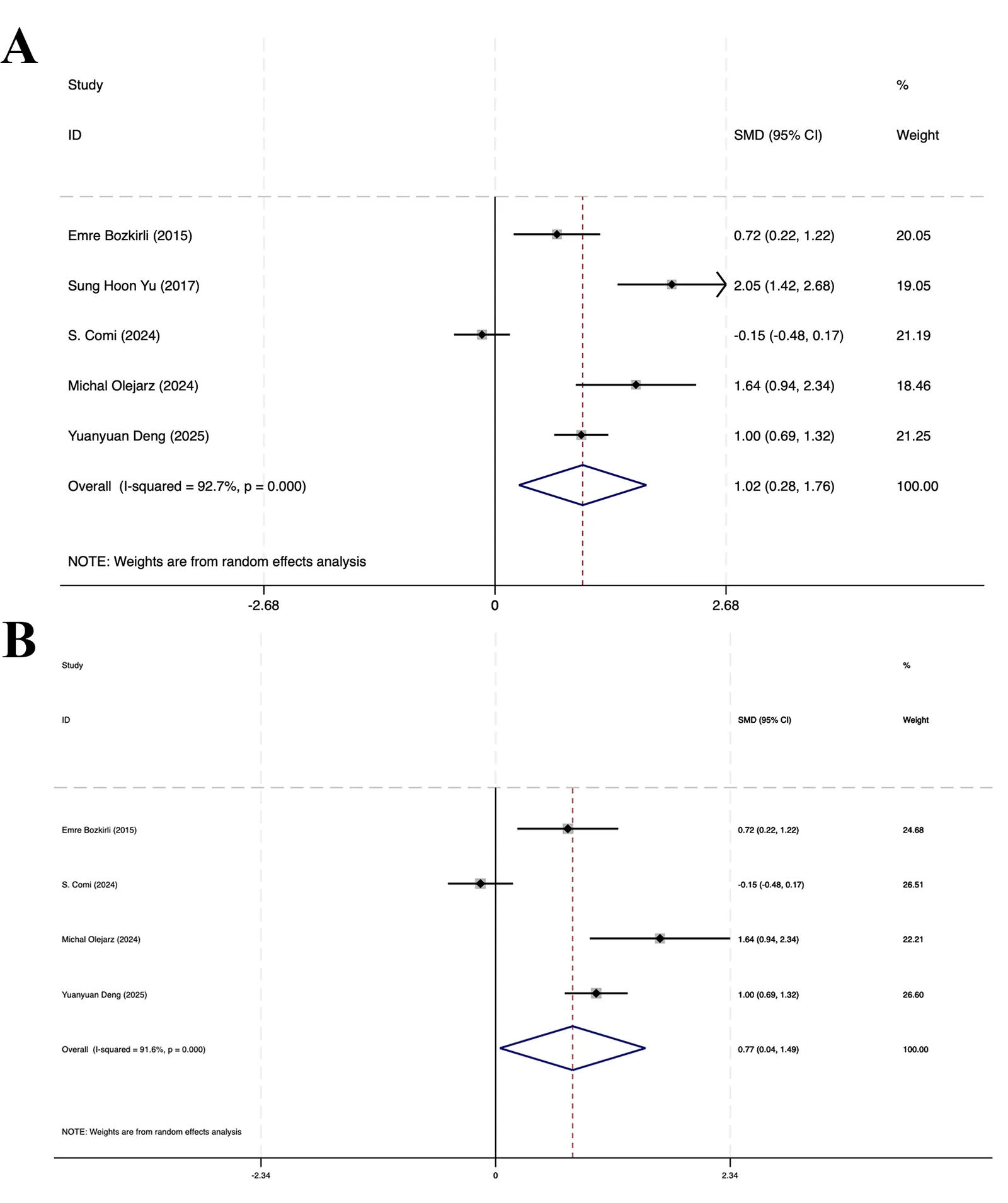
**(A)** Forest plot for the meta-analysis comparing serum IgG4 levels between patients with GO and those suffering from GD without ophthalmopathy; **(B)** Forest plot for the sensitivity analysis comparing patients with GO and those suffering from GD without ophthalmopathy after excluding the study by Yu et al. (19).

Serum IgG4 levels among individuals with GO in comparison with healthy controls, and among patients with GO compared with those with GD, were evaluated in five studies each. High heterogeneity was detected among the studies for both outcome indicators (I² = 83.5%; I² = 92.7%, respectively). Accordingly, a random-effects model was adopted. The meta-analysis revealed that the pooled effect sizes were: SMD = 1.260 (95% CI: 0.681, 1.839; *p* < 0.05) and SMD = 1.018 (95% CI: 0.278, 1.758; *p* < 0.05), respectively, supporting that IgG4 levels in the GO group were pronouncedly higher than those in both the normal control group and the GD group (**Figures 2A**, **3A**).

The sensitivity analysis, conducted by sequentially eliminating an individual study, demonstrated that after the exclusion of the study by Sung Hoon Yu et al. (19) from the comparison between GO and healthy controls, the heterogeneity was reduced (I² = 0.0%). However, this exclusion did not materially alter the overall pooled estimates, suggesting that the findings were stable (**Figures 2B**, **3B**).

### 3.2 Incidence rate and risk of GO associated with low and high IgG4 levels

**Figure 4:**
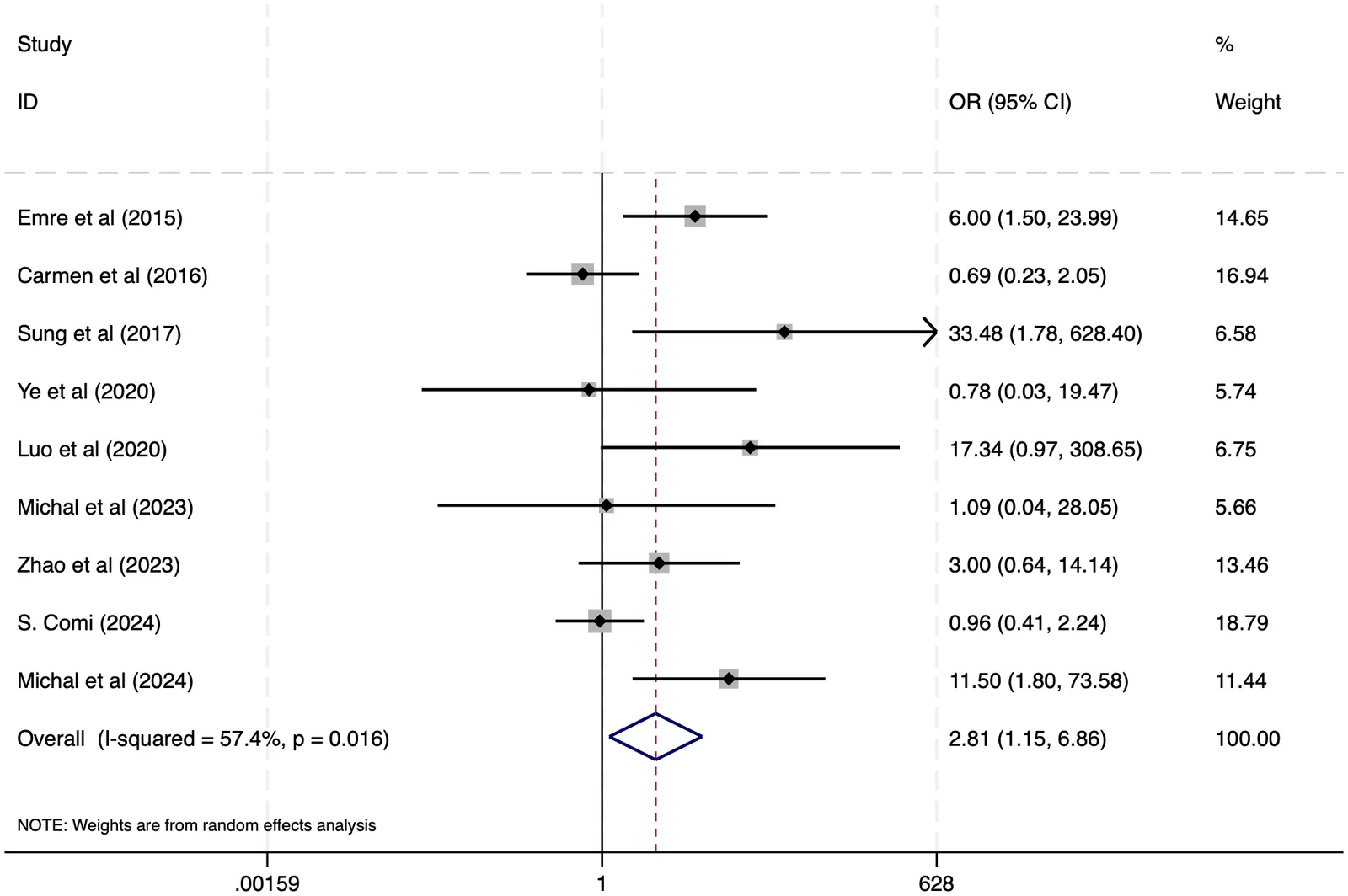
Forest plot for the meta-analysis comparing serum IgG4 levels between patients with GO and those without GO.

The incidence rate of GO in individuals with a high IgG4 level versus those with a low IgG4 level was assessed in nine studies. Substantial heterogeneity was detected across these studies (I² = 90.7%). Accordingly, a random-effects model was employed. The meta-analysis revealed a higher incidence rate of GO in individuals with a high IgG4 level compared with those with a low IgG4 level (OR = 2.808; 95% CI: 1.150, 6.859; *p* < 0.05) (**Figure 4**).

**Figure 5:**
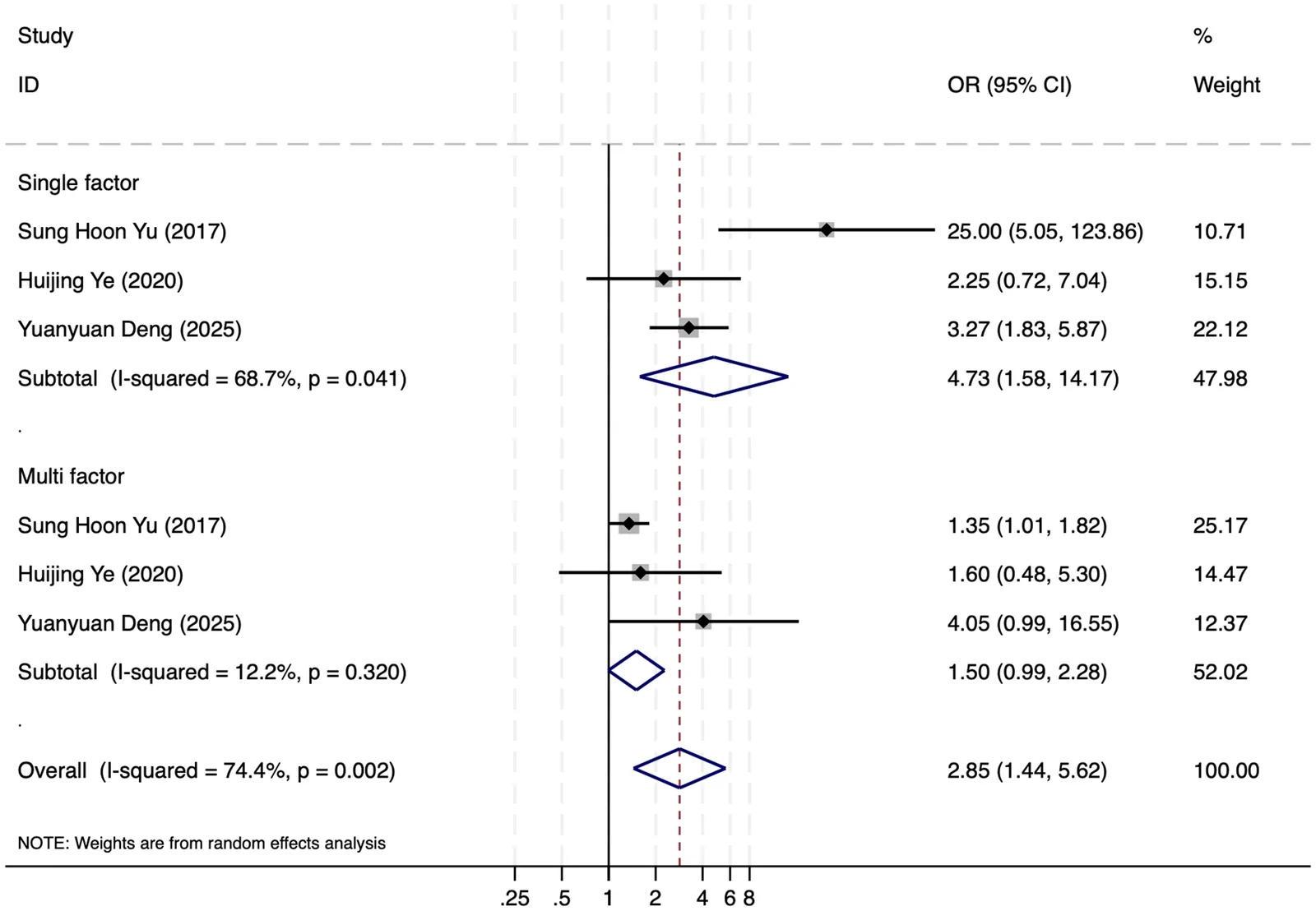
Forest plot for the univariable and multivariable meta-analyses of the association between serum IgG4 levels and GO risk.

This finding was corroborated by the risk data analyses. In the univariable analysis, a significantly elevated risk of GO was associated with a higher IgG4 level (ES = 4.735; 95% CI: 1.582, 14.171; I² = 68.7%; p = 0.005). In the multivariable analysis, although a heightened IgG4 level was associated with an increased risk of GO, this association failed to reach statistical significance (ES = 1.504; 95% CI: 0.994, 5.616; I² = 12.2%; p = 0.053). The source of heterogeneity primarily stemmed from the univariable analysis. However, the overall results were not influenced (**Figure 5**).

### 3.3 IgG4 levels in active and inactive GO

**Figure 6:**
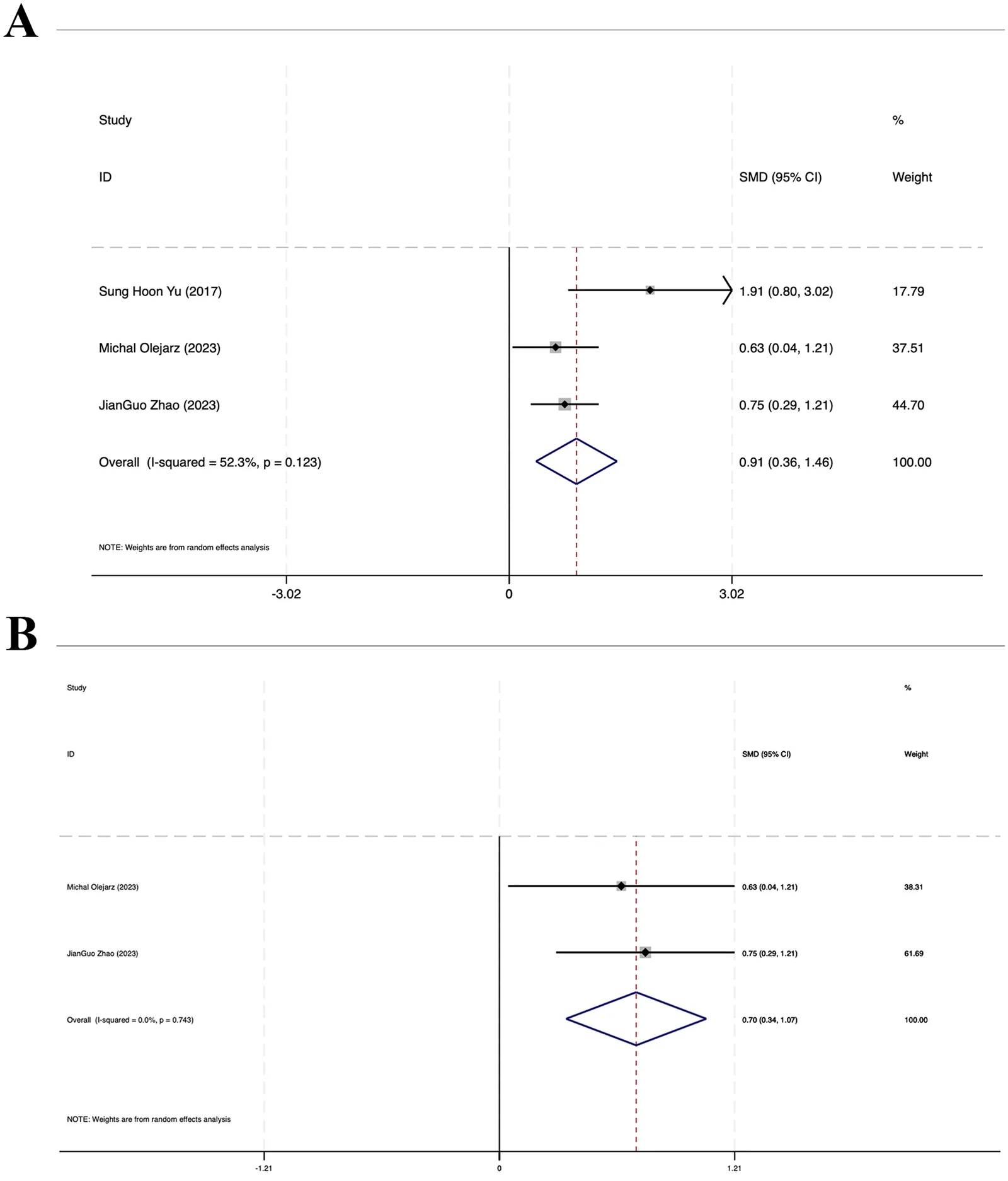
**(A)** Forest plot for the meta-analysis comparing serum IgG4 levels between patients with active GO and those with inactive GO; **(B)** Forest plot for the sensitivity analysis comparing patients with active GO and those with inactive GO after excluding the study by Yu et al. (19).

Serum IgG4 levels among individuals with active GO in comparison to those with inactive GO were evaluated in three studies. Moderate heterogeneity was observed (I² = 52.3%). The meta-analysis indicated that IgG4 levels in the active GO group were significantly higher than those in the inactive GO group (SMD = 0.911 (95% CI: 0.363, 1.460; *p* < 0.05) (**Figure 6A**).

The sensitivity analysis demonstrated that after the removal of the study by Sung Hoon Yu et al. (19), the heterogeneity was reduced to I² = 0.0%, suggesting that the heterogeneity primarily stemmed from the study. However, this exclusion did not substantially change the overall pooled estimate, signifying that the findings were stable (**Figure 6B**).

### 3.4 Subgroup analysis

Further subgroup analyses were implemented based on geographical region, study design, and measurement method. In the studies conducted in Asia and the Middle East, IgG4 levels were pronouncedly elevated in the GO group in comparison with the GD group, and a higher IgG4 level was linked to a heightened risk of GO (*p* < 0.05). Among the cross-sectional studies, IgG4 levels were markedly higher in the GO group in comparison to both the normal control group and the GD group, and individuals with a higher IgG4 level tended to develop GO (*p* < 0.05). In studies employing an immunoturbidimetric assay, a higher IgG4 level was more associated with the onset of GO (*p* < 0.05). These findings suggested that the selection of measurement method might influence the observed association with GO. The results are detailed in **Table 2** and **Supplementary Material 2**.

**Table 2:** Subgroup analysis of factors influencing the association between serum IgG4 levels and GO.

Outcome	SMD and 95% CI	*P* Value	I²(%)
Study region			
GO vs GD	1.018(0.278, 1.758)	0.007	92.7
GO vs Normal	1.260(0.681, 1.839)	0	83.5
Active GO vs Inactive GO	0.911(0.363, 1.460)	0.001	52.3
High IgG4 vs Lower IgG4	2.808(1.150, 6.859)	0.023	57.4
Study design			
GO vs GD	1.038(-0.003, 2.078)	0.051	93.9
GO vs Normal	1.260(0.681, 1.839)	0	83.5
High IgG4 vs Lower IgG4	2.808(1.150, 6.859)	0.023	57.4
Assay method			
GO vs GD	1.018(0.278, 1.758)	0.007	92.7
GO vs Normal	1.260(0.681, 1.839)	0	83.5
Active GO vs Inactive GO	0.911(0.363, 1.460)	0.001	52.3
High IgG4 vs Lower IgG4	2.808(1.150, 6.859)	0.023	57.4

### 3.5 Publication bias

**Figure 7:**
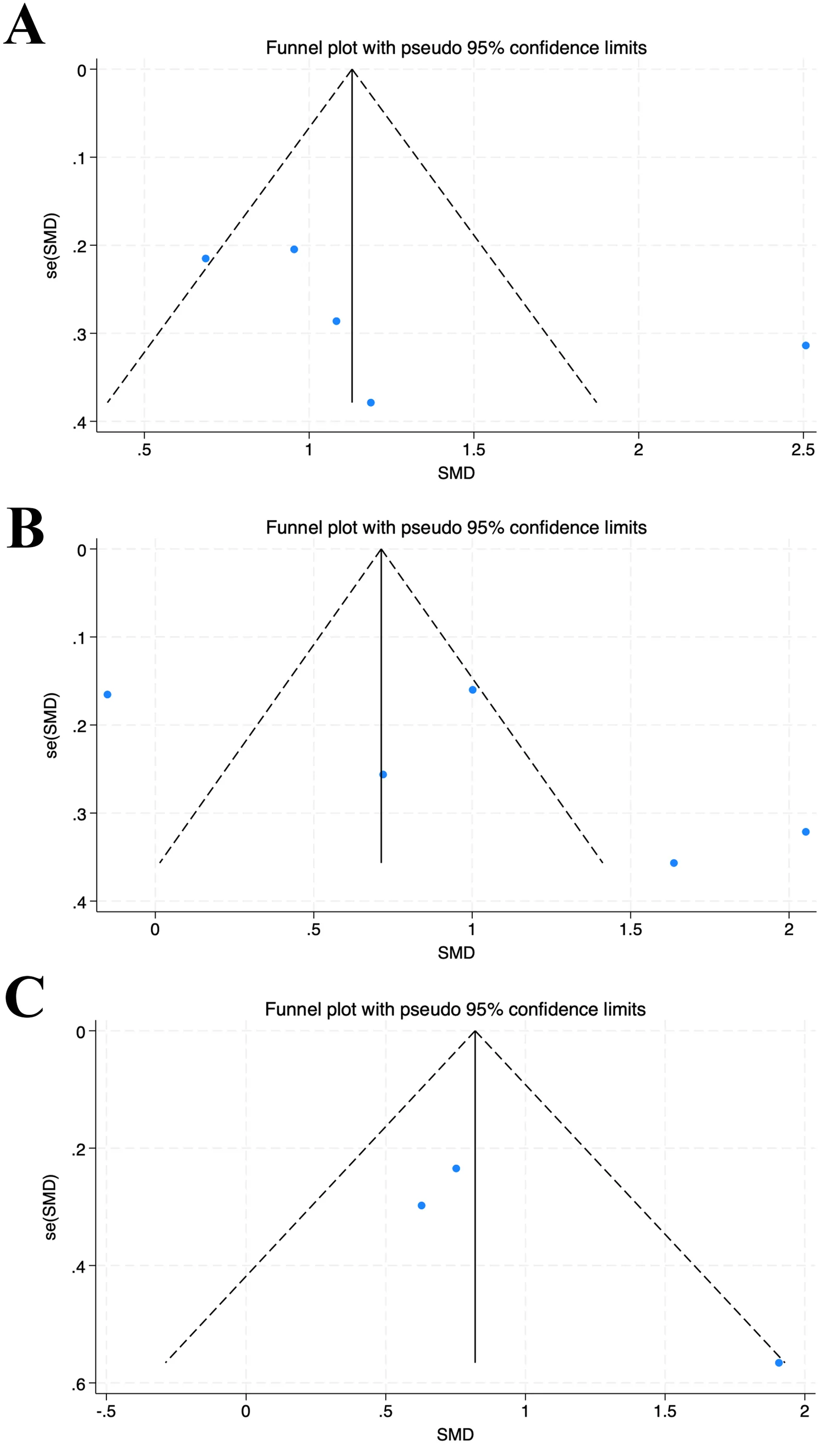
**(A)** Publication bias analysis of serum IgG4 levels between patients with GO and healthy controls; **(B)** Publication bias analysis of serum IgG4 levels between patients with GO and those suffering from GD without ophthalmopathy; **(C)** Publication bias analysis of serum IgG4 levels between patients with active GO and those with inactive GO.

Publication bias was assessed for the included studies. The funnel plot was roughly symmetrical, signifying an absence of publication bias (**Figure 7**). The Egger’s test further identified *p*>0.05.

## 4 Discussion

This study, through a systematic review and meta-analysis, probed into the association of serum IgG4 level with GO. The principal findings are multifaceted. Firstly, in the comparisons between case-control studies, serum IgG4 levels were observed to be pronouncedly higher among individuals with GO than in both healthy controls and patients suffering from GD without ophthalmopathy. Although substantial heterogeneity was detected among the included studies, the robustness of this result was confirmed by the random-effects model and through the sensitivity analyses. This suggested a specific association between elevated IgG4 and GO. Secondly, concerning GO risk, higher IgG4 levels were observed to significantly increase the risk of developing GO. This role as a potential risk factor was further supported by the univariable analysis. Despite the fact that the association failed to reach statistical significance in the multivariable analysis, the trend was consistent, suggesting that IgG4 might be an important risk indicator that was potentially modulated by other clinical variables. Meta-regression analysis indicated that factors such as study region, study design, and IgG4 measurement method did not affect the overall results. This study confirmed through meta-analysis that IgG4 levels in patients with active GO were higher than those in patients with inactive GO. All included studies employed the clinical activity score (CAS) for disease activity assessment, ensuring consistency in assessment criteria and reducing clinical heterogeneity, suggesting that IgG4 levels may be associated with disease activity and severity. The subgroup analyses revealed that the association between IgG4 and GO was more pronounced among studies conducted in Asian and Middle Eastern populations, those employing a cross-sectional design, and those utilizing an immunoturbidimetric assay. This finding suggested that it was important to standardize detection methods and pay attention to population characteristics in clinical settings. The assessment of publication bias for all the primary outcomes revealed that no significant bias was detected, thereby enhancing the reliability of the aforementioned findings.

An IgG4 level was specifically elevated among patients with GO, which prompted a re-examination of the immunological nature of GO. Conventionally, the pathogenesis of GO has focused primarily on the autoimmune response directed against the TSH receptor (TSHR), with a central role attributed to TRAb (30–33). Nevertheless, this study found that serum IgG4 levels were significantly higher among individuals with GO than in those suffering from GD without ophthalmopathy. An elevation in IgG4 level is not simply parallel to the systemic state of thyroid autoimmunity but is specifically associated with the pathological processes occurring within the orbital tissues (34–36).

This observation leads to a hypothesis that a local immune microenvironment shift, from a Th1-dominated inflammatory response towards a Th2-predominant immune reaction, may occur in a subset of patients with GO (37–40). Th2-type cytokines (e.g., IL-4 and IL-13) are recognized as key drivers in inducing immunoglobulin class switching and the subsequent production of IgG4 in B cells (9, 13, 41, 42). Consequently, lymphocytes infiltrating the orbital tissues may secrete these Th2-type cytokines, thereby promoting the local or systemic generation of substantial quantities of IgG4 by antigen-specific B cells (43–45). Should this hypothesis be confirmed, it would significantly enhance the understanding of the immunological complexity underlying GO. This suggests that GO is not merely a single-autoantibody disease, but rather an intricate condition involving multiple cell types and factors, potentially characterized by distinct immune phenotypes.

Building on this premise, the finding of a significant positive association between an increased IgG4 level and a risk of developing GO elevates IgG4 from a simple associated marker to a potential risk predictor. This implies that measuring serum IgG4 levels might help identify individuals at an elevated risk for GO, thereby providing an early warning signal for early intervention and close follow-up. It is noteworthy that the association between IgG4 and GO risk was attenuated and failed to reach statistical significance in the multivariable analysis. This attenuation precisely reflects the complexity inherent in clinical settings. This finding indicates that IgG4 may not function as a completely independent risk factor, but rather interacts with established risk factors such as smoking, high TRAb level, and disease duration (5). TRAb at high concentration possibly more potently activates the TSHR on orbital fibroblasts (46, 47), thereby creating a local environment more conducive to Th2 polarization and IgG4 production. Therefore, in the future, prospective studies should evaluate IgG4 within a broader network of risk factors and develop multivariable prediction models to more precisely quantify the GO risk for an individual patient.

**Figure 8:**
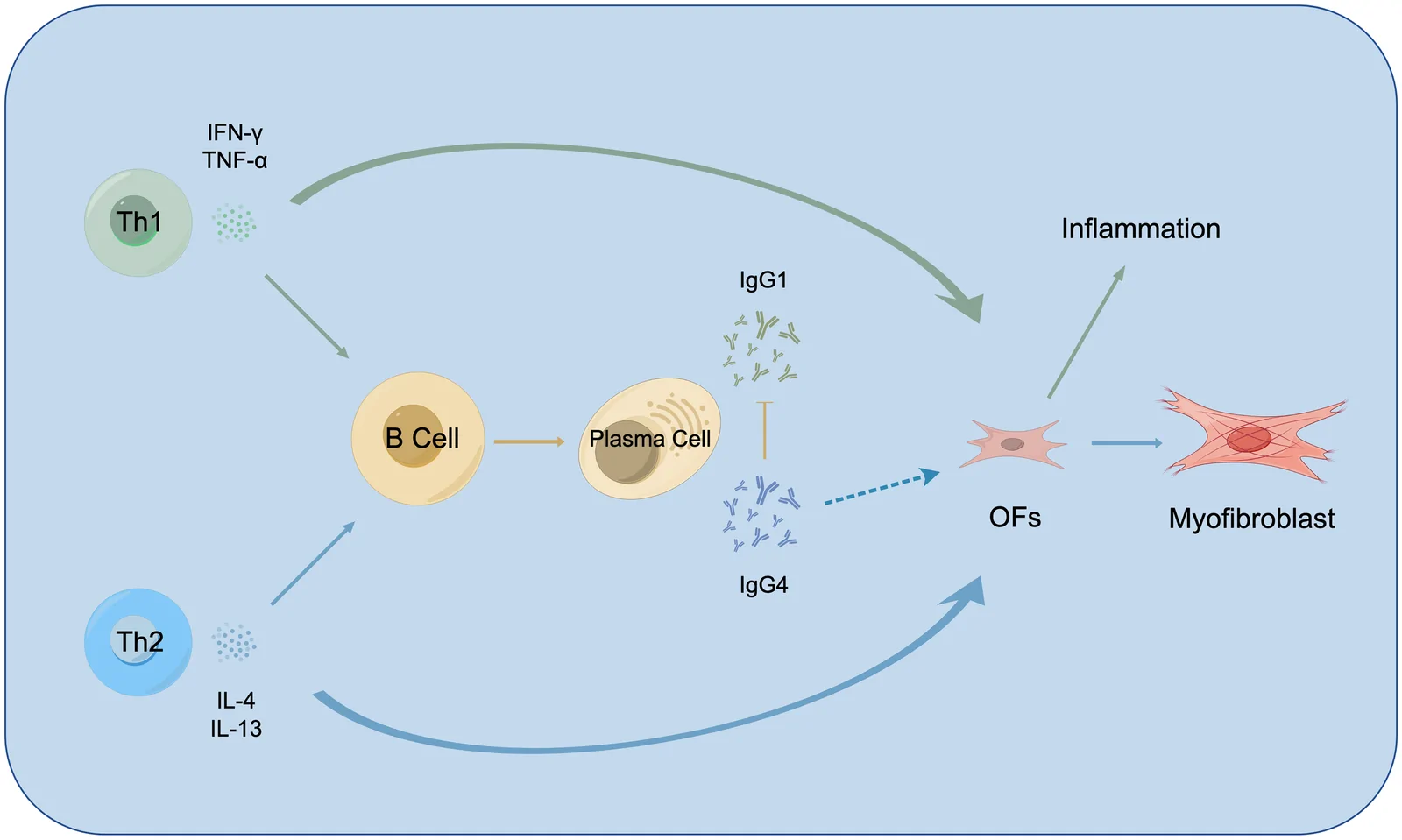
Th1 and Th2 immunopathways in GO The Th1-driven inflammatory axis (left/upper): Th1 cells secrete IFN-γ and TNF-α, which promote B cell differentiation into plasma cells producing pathogenic IgG1 antibodies. These cytokines also activate OFs (orbital fibroblasts) and perpetuate local inflammation, contributing to the acute active phase of GO. The Th2-driven fibrotic axis (right/lower): Th2 cells secrete IL-4 and IL-13, which drive B cell differentiation into plasma cells producing pathogenic IgG4 antibodies. Concurrently, IL-4 and IL-13 stimulate OFs to transdifferentiate into myofibroblasts, leading to excessive extracellular matrix production, tissue fibrosis, and irreversible orbital remodeling during the chronic phase of GO. IFN-γ, interferon-gamma; TNF-α, tumor necrosis factor-alpha; IL, interleukin; IgG, immunoglobulin G; OFs, orbital fibroblasts. (This figure is drawn using Figdraw).

This study revealed an association of serum IgG4 levels with GO activity. Active GO, featuring inflammation, edema, and cellular infiltration of the orbital tissues, serves as the primary indication for immunosuppressive therapy (48–50). In this study, IgG4 levels were pronouncedly higher among individuals with active GO in comparison with those with inactive GO, suggesting that IgG4 might be involved in the processes of chronic inflammation and fibrosis within the orbit. The underlying mechanisms may be multifaceted. Although IgG4 is known for its weak effector functions, it is not entirely inert. IgG4 may potentially stimulate the proliferation and activation of orbital fibroblasts through repeated engagement with putative autoantigens on their surface, thereby promoting glycosaminoglycan secretion and mediating tissue remodeling. Furthermore, the unique ability of IgG4 to undergo Fab-arm exchange results in bispecific antibodies (10, 12), possibly interfering with the function of pathogenic IgG1 antibodies, thereby serving as an immunomodulatory factor (51–56). However, within the specific context of GO, it is worth investigating whether this modulatory function becomes dysregulated, paradoxically contributing to the pathological process. In summary, treating serum IgG4 level as an auxiliary biomarker for evaluating GO activity, and using serum IgG4 level in conjunction with the CAS, holds promise for reducing the subjectivity of clinical evaluation and providing more objective laboratory evidence for initiating or adjusting immunotherapy. Details are available in **Figure 8**.

A series of methodological approaches was employed to explore the substantial heterogeneity observed in this meta-analysis. In view of the substantial heterogeneity in this analysis, we performed an in-depth investigation through sensitivity analyses and meta-regression. The sensitivity analysis indicated that although individual studies contributed to the observed heterogeneity, sequentially excluding them did not change the direction or significance of the overall effect estimates. This strongly supports the robustness of the core findings. Meta-regression results showed that factors such as study region, study design, and measurement methods did not influence the overall results. Based on previous literature, we speculate that the sources of heterogeneity may be clinical or biological factors that have not been adequately measured, including: prior treatment history (e.g., glucocorticoids or radioactive iodine therapy), endocrine and autoimmune abnormalities (e.g., thyroid dysfunction, elevated autoantibody levels), psychosocial factors (e.g., ethnic and cultural differences, psychological stress, socioeconomic status), metabolic and nutritional status (e.g., obesity, hypercholesterolemia, selenium or vitamin D deficiency), and smoking exposure (including active smoking and second-hand smoke). Moreover, GO itself may have as-yet-unclear endotypes, and the combined effect of these factors may significantly influence serum IgG4 levels (57).

Notably, the subgroup analyses suggested that the selection of IgG4 measurement method might influence the detection of its association with GO. This finding highlights that it is imperative to standardize the detection of serum IgG4 and establish uniform cutoff values in both research and clinical practice in the future. All ten included studies explicitly reported their adopted cutoff values: seven (70%) used the internationally accepted IgG4 ≥135 mg/dL as the positive threshold, one used an IgG4/IgG ratio ≥8%, and the remaining two employed the upper quartile of the study population (IgG4 ≥237.52 mg/dL and ≥86.4 mg/dL, respectively) as customized cutoff values. Despite heterogeneity in cutoff selection, most studies adhered to the currently recommended 135 mg/dL standard (58), providing a primary basis for clinical reference. To investigate the potential impact of inconsistent cutoff values on the robustness of the results, we performed analyses based on the pooled data from the included studies. First, a sensitivity analysis restricted to the 7 studies using the 135 mg/dL cutoff yielded a pooled effect estimate and 95% CI consistent in direction with the overall analysis, with no marked increase in heterogeneity, suggesting good consistency of the association signal under this cutoff value. Second, since only 3 studies used non-standard cutoff values with widely differing definitions, a meaningful subgroup meta-regression or threshold-effect analysis was not feasible. Therefore, the current pooled data are insufficient to derive, through statistical methods, a new cutoff superior to the existing consensus. Based on the available evidence, we recommend that in clinical practice and future confirmatory studies, the widely validated cutoff of IgG4 ≥135 mg/dL should be preferentially used, with clear documentation of the assay kit and calibrator source to reduce methodological bias. For studies using non-standard cutoff values, we suggest that in the future, multi-center, large-sample studies be performed to assess the applicability of optimal thresholds across different populations and disease activity states, thereby promoting the clinical standardization of this biomarker.

Elevated IgG4 with orbital inflammation is not unique to GO and requires differentiation from IgG4-related ophthalmic disease (IgG4-ROD). The key points are as follows: Clinically, GO is typically accompanied by thyroid dysfunction and positive TRAb, whereas IgG4-ROD typically presents with normal thyroid function and is frequently associated with a history of asthma and progressive orbital disease. On imaging, GO typically shows extraocular muscle involvement with tendon-sparing and belly enlargement, more commonly affecting the inferior and medial rectus muscles, while IgG4-ROD is characterized by disproportionate enlargement of the lateral rectus muscle and infraorbital nerve thickening. Serologically, IgG4 ≥135 mg/dL is a diagnostic threshold for IgG4-ROD; however, some patients with GO may also have elevated IgG4 without meeting other diagnostic criteria. Definitive diagnosis relies on pathological biopsy, which reveals infiltration by IgG4-positive plasma cells with an IgG4/IgG ratio >40%. In addition, the two diseases may occur sequentially or simultaneously, suggesting possible overlap in immunopathological mechanisms (54–56, 59–61). Therefore, the results of this meta-analysis should be interpreted with caution because the original studies may have included unrecognized IgG4-ROD cases, which could explain the between-study heterogeneity in effect sizes. In the future, additional studies are warranted to stratify patients with GO by serum IgG4 levels and to incorporate comprehensive imaging and pathological examinations, in order to more accurately assess the association between IgG4 and GO.

Although insightful conclusions have been reached through rigorous analysis in this study, several limitations should be interpreted cautiously. Firstly, the majority of the included studies employed case-control or cross-sectional designs, which essentially failed to establish a causal relationship between IgG4 and GO. It could not be determined whether elevated IgG4 levels contributed to the onset and progression of GO, or whether the active pathological process of GO induced a reactive increase in IgG4. Secondly, data on individual patients were not available for this analysis, rendering it difficult to precisely control for all potential confounders, such as smoking status, level of thyroid function control, and specific treatment regimens. These unmeasured variables might represent latent sources of heterogeneity. Finally, the current body of research investigating the relationship between IgG4 and GO remains limited in quantity. Particularly, the analysis comparing active and inactive GO was based on only three studies. Thus, these specific findings should be interpreted with caution. The association between IgG4 levels and GO disease activity is still a preliminary finding, and its clinical value as an activity biomarker warrants to be further validated through larger-sample, well-designed prospective studies. In addition, the literature search in this study was restricted to English-language SCI-indexed journals, primarily because such journals enforce rigorous peer-review processes, which can effectively ensure the academic quality and data reliability of the included studies and also facilitate consistency in data extraction and quality assessment. Nevertheless, this strategy may introduce language bias, potentially omitting high-quality studies published in non-English journals, thereby affecting the comprehensiveness and generalizability of our findings.

This study identifies several significant directions for research in the future. A primary priority is implementing large-scale, multicenter prospective cohort studies with prolonged patient follow-up. Such studies should dynamically monitor serum IgG4 levels and other immunological parameters to elucidate the evolutionary pattern of IgG4 throughout the natural course of GO and to determine the predictive value of IgG4. At the basic science level, the specific pathophysiological role of IgG4 in GO needs to be further explored. Key avenues for investigation include identifying the target antigen(s) recognized by IgG4 within orbital tissues, studying the direct effects of IgG4 on the function of orbital fibroblasts, and validating its pathogenicity *in vivo* via animal models. A particularly compelling hypothesis to be tested is whether a distinct ‘IgG4-related GO’ subtype exists (62).

## 5 Conclusion

To summarize, this study has found, with robust evidence, a close association of serum IgG4 level with the presence, risk, and activity of GO. In existing research, IgG4 has been regarded as a particular immune molecule to investigate other fibrotic inflammatory diseases. In this study, IgG4 is introduced into the research on GO. This approach has not only opened a new perspective in GO research but has also helped revise and refine existing theories. Although the directionality of the association and underlying mechanisms remain to be fully elucidated before clinical application, this study represents a significant milestone. This study provides compelling support for the underlying value of integrating IgG4 into a multidimensional evaluation system for GO. Moreover, it illuminates a promising new direction for ultimately unraveling the complex immunological basis of GO and paving the way towards a future of more precise and effective individualized therapy.

## Data Availability

The original contributions presented in the study are included in the article/**Supplementary Material**. Further inquiries can be directed to the corresponding author.
