# Supplementary material for: Association between serum immunoglobulin G4 levels and Graves’ ophthalmopathy: a meta-analysis
Source: Front Endocrinol (Lausanne). 2026 Sep 10;17:1837951. doi: 10.3389/fendo.2026.1837951 (PMC13600816; doi:10.3389/fendo.2026.1837951)
Supplement: Supplementary file 1 [file Supplementaryfile1.docx]

**Supplementary material 1.** Search Strategy

**Search Strategy for PubMed**

Graves Ophthalmopathy

"Congestive Ophthalmopathies" OR "Congestive Ophthalmopathy" OR "Disease, Graves Eye" OR "Disease, Thyroid Eye" OR "dysthyroid eye disease" OR "Dysthyroid Ophthalmopathies" OR "Dysthyroid Ophthalmopathy" OR "dysthyroid orbitopathy" OR "Edematous Ophthalmopathies" OR "Edematous Ophthalmopathy" OR "endocrine ophthalmopathy" OR "endocrine orbitopathy" OR "Eye Disease, Graves" OR "Eye Disease, Thyroid" OR "Graves Eye Disease" OR "Graves ophthalmopathy" OR "Graves Orbitopathy" OR "Infiltrative Ophthalmopathies" OR "Infiltrative Ophthalmopathy" OR "Myopathic Ophthalmopathies" OR "Myopathic Ophthalmopathy" OR "ophthalmic Graves disease" OR "Ophthalmopathies, Thyroid Associated" OR "Ophthalmopathy, Congestive" OR "Ophthalmopathy, Dysthyroid" OR "Ophthalmopathy, Edematous" OR "ophthalmopathy, endocrine" OR "Ophthalmopathy, Graves" OR "Ophthalmopathy, Infiltrative" OR "Ophthalmopathy, Myopathic" OR "Ophthalmopathy, Thyroid Associated" OR "Orbitopathy, Graves" OR "thyroid associated eye disease" OR "Thyroid Associated Ophthalmopathies" OR "Thyroid Associated Ophthalmopathy" OR "thyroid associated orbitopathy" OR "Thyroid Eye Disease" OR "Thyroid Eye Diseases" OR "thyroid ophthalmopathy" OR "thyroid orbitopathy" OR "thyroid related orbitopathy"

Immunoglobulin G4

"gamma g4" OR "igg 4" OR "IgG4" OR "IgG4 level" OR "IgG4 Serum" OR "immunoglobulin 4" OR "Immunoglobulin G4 "

(#1 OR #2) AND (#3 OR #4)

**Search Strategy for Embase**

('Congestive Ophthalmopathies' OR 'Congestive Ophthalmopathy' OR 'Disease, Graves Eye' OR 'Disease, Thyroid Eye' OR 'dysthyroid eye disease' OR 'Dysthyroid Ophthalmopathies' OR 'Dysthyroid Ophthalmopathy' OR 'dysthyroid orbitopathy' OR 'Edematous Ophthalmopathies' OR 'Edematous Ophthalmopathy' OR 'endocrine ophthalmopathy' OR 'endocrine orbitopathy' OR 'Eye Disease, Graves' OR 'Eye Disease, Thyroid' OR 'Graves Eye Disease' OR 'Graves ophthalmopathy' OR 'Graves Orbitopathy' OR 'Infiltrative Ophthalmopathies' OR 'Infiltrative Ophthalmopathy' OR 'Myopathic Ophthalmopathies' OR 'Myopathic Ophthalmopathy' OR 'ophthalmic Graves disease' OR 'Ophthalmopathies, Thyroid Associated' OR 'Ophthalmopathy, Congestive' OR 'Ophthalmopathy, Dysthyroid' OR 'Ophthalmopathy, Edematous' OR 'ophthalmopathy, endocrine' OR 'Ophthalmopathy, Graves' OR 'Ophthalmopathy, Infiltrative' OR 'Ophthalmopathy, Myopathic' OR 'Ophthalmopathy, Thyroid Associated' OR 'Orbitopathy, Graves' OR 'thyroid associated eye disease' OR 'Thyroid Associated Ophthalmopathies' OR 'Thyroid Associated Ophthalmopathy' OR 'thyroid associated orbitopathy' OR 'Thyroid Eye Disease' OR 'Thyroid Eye Diseases' OR 'thyroid ophthalmopathy' OR 'thyroid orbitopathy' OR 'thyroid related orbitopathy'):ti,ab,kw

'endocrine ophthalmopathy'/exp

('gamma g4' OR 'igg 4' OR 'IgG4' OR 'IgG4 level' OR 'IgG4 Serum' OR 'immunoglobulin 4' OR 'Immunoglobulin G4 '):ti,ab,kw

'immunoglobulin G4'/exp

**Search Strategy for Cochrane Library**

("Congestive Ophthalmopathies") OR ("Congestive Ophthalmopathy") OR ("Disease, Graves Eye") OR ("Disease, Thyroid Eye") OR ("dysthyroid eye disease") OR ("Dysthyroid Ophthalmopathies") OR ("Dysthyroid Ophthalmopathy") OR ("dysthyroid orbitopathy") OR ("Edematous Ophthalmopathies") OR ("Edematous Ophthalmopathy") OR ("endocrine ophthalmopathy") OR ("endocrine orbitopathy") OR ("Eye Disease, Graves") OR ("Eye Disease, Thyroid") OR ("Graves Eye Disease") OR ("Graves ophthalmopathy") OR ("Graves Orbitopathy") OR ("Infiltrative Ophthalmopathies") OR ("Infiltrative Ophthalmopathy") OR ("Myopathic Ophthalmopathies") OR ("Myopathic Ophthalmopathy") OR ("ophthalmic Graves disease") OR ("Ophthalmopathies, Thyroid Associated") OR ("Ophthalmopathy, Congestive") OR ("Ophthalmopathy, Dysthyroid") OR ("Ophthalmopathy, Edematous") OR ("ophthalmopathy, endocrine") OR ("Ophthalmopathy, Graves") OR ("Ophthalmopathy, Infiltrative") OR ("Ophthalmopathy, Myopathic") OR ("Ophthalmopathy, Thyroid Associated") OR ("Orbitopathy, Graves") OR ("thyroid associated eye disease") OR ("Thyroid Associated Ophthalmopathies") OR ("Thyroid Associated Ophthalmopathy") OR ("thyroid associated orbitopathy") OR ("Thyroid Eye Disease") OR ("Thyroid Eye Diseases") OR ("thyroid ophthalmopathy") OR ("thyroid orbitopathy") OR ("thyroid related orbitopathy")

("gamma g4") OR ("igg 4") OR ("IgG4") OR ("IgG4 level") OR ("IgG4 Serum") OR ("immunoglobulin 4") OR ("Immunoglobulin G4")

**Search Strategy for WoS**

(TI=(("Congestive Ophthalmopathies") OR ("Congestive Ophthalmopathy") OR ("Disease, Graves Eye") OR ("Disease, Thyroid Eye") OR ("dysthyroid eye disease") OR ("Dysthyroid Ophthalmopathies") OR ("Dysthyroid Ophthalmopathy") OR ("dysthyroid orbitopathy") OR ("Edematous Ophthalmopathies") OR ("Edematous Ophthalmopathy") OR ("endocrine ophthalmopathy") OR ("endocrine orbitopathy") OR ("Eye Disease, Graves") OR ("Eye Disease, Thyroid") OR ("Graves Eye Disease") OR ("Graves ophthalmopathy") OR ("Graves Orbitopathy") OR ("Infiltrative Ophthalmopathies") OR ("Infiltrative Ophthalmopathy") OR ("Myopathic Ophthalmopathies") OR ("Myopathic Ophthalmopathy") OR ("ophthalmic Graves disease") OR ("Ophthalmopathies, Thyroid Associated") OR ("Ophthalmopathy, Congestive") OR ("Ophthalmopathy, Dysthyroid") OR ("Ophthalmopathy, Edematous") OR ("ophthalmopathy, endocrine") OR ("Ophthalmopathy, Graves") OR ("Ophthalmopathy, Infiltrative") OR ("Ophthalmopathy, Myopathic") OR ("Ophthalmopathy, Thyroid Associated") OR ("Orbitopathy, Graves") OR ("thyroid associated eye disease") OR ("Thyroid Associated Ophthalmopathies") OR ("Thyroid Associated Ophthalmopathy") OR ("thyroid associated orbitopathy") OR ("Thyroid Eye Disease") OR ("Thyroid Eye Diseases") OR ("thyroid ophthalmopathy") OR ("thyroid orbitopathy") OR ("thyroid related orbitopathy")) OR AB=(("Congestive Ophthalmopathies") OR ("Congestive Ophthalmopathy") OR ("Disease, Graves Eye") OR ("Disease, Thyroid Eye") OR ("dysthyroid eye disease") OR ("Dysthyroid Ophthalmopathies") OR ("Dysthyroid Ophthalmopathy") OR ("dysthyroid orbitopathy") OR ("Edematous Ophthalmopathies") OR ("Edematous Ophthalmopathy") OR ("endocrine ophthalmopathy") OR ("endocrine orbitopathy") OR ("Eye Disease, Graves") OR ("Eye Disease, Thyroid") OR ("Graves Eye Disease") OR ("Graves ophthalmopathy") OR ("Graves Orbitopathy") OR ("Infiltrative Ophthalmopathies") OR ("Infiltrative Ophthalmopathy") OR ("Myopathic Ophthalmopathies") OR ("Myopathic Ophthalmopathy") OR ("ophthalmic Graves disease") OR ("Ophthalmopathies, Thyroid Associated") OR ("Ophthalmopathy, Congestive") OR ("Ophthalmopathy, Dysthyroid") OR ("Ophthalmopathy, Edematous") OR ("ophthalmopathy, endocrine") OR ("Ophthalmopathy, Graves") OR ("Ophthalmopathy, Infiltrative") OR ("Ophthalmopathy, Myopathic") OR ("Ophthalmopathy, Thyroid Associated") OR ("Orbitopathy, Graves") OR ("thyroid associated eye disease") OR ("Thyroid Associated Ophthalmopathies") OR ("Thyroid Associated Ophthalmopathy") OR ("thyroid associated orbitopathy") OR ("Thyroid Eye Disease") OR ("Thyroid Eye Diseases") OR ("thyroid ophthalmopathy") OR ("thyroid orbitopathy") OR ("thyroid related orbitopathy")) OR AK=(("Congestive Ophthalmopathies") OR ("Congestive Ophthalmopathy") OR ("Disease, Graves Eye") OR ("Disease, Thyroid Eye") OR ("dysthyroid eye disease") OR ("Dysthyroid Ophthalmopathies") OR ("Dysthyroid Ophthalmopathy") OR ("dysthyroid orbitopathy") OR ("Edematous Ophthalmopathies") OR ("Edematous Ophthalmopathy") OR ("endocrine ophthalmopathy") OR ("endocrine orbitopathy") OR ("Eye Disease, Graves") OR ("Eye Disease, Thyroid") OR ("Graves Eye Disease") OR ("Graves ophthalmopathy") OR ("Graves Orbitopathy") OR ("Infiltrative Ophthalmopathies") OR ("Infiltrative Ophthalmopathy") OR ("Myopathic Ophthalmopathies") OR ("Myopathic Ophthalmopathy") OR ("ophthalmic Graves disease") OR ("Ophthalmopathies, Thyroid Associated") OR ("Ophthalmopathy, Congestive") OR ("Ophthalmopathy, Dysthyroid") OR ("Ophthalmopathy, Edematous") OR ("ophthalmopathy, endocrine") OR ("Ophthalmopathy, Graves") OR ("Ophthalmopathy, Infiltrative") OR ("Ophthalmopathy, Myopathic") OR ("Ophthalmopathy, Thyroid Associated") OR ("Orbitopathy, Graves") OR ("thyroid associated eye disease") OR ("Thyroid Associated Ophthalmopathies") OR ("Thyroid Associated Ophthalmopathy") OR ("thyroid associated orbitopathy") OR ("Thyroid Eye Disease") OR ("Thyroid Eye Diseases") OR ("thyroid ophthalmopathy") OR ("thyroid orbitopathy") OR ("thyroid related orbitopathy"))) AND (TI=(("gamma g4") OR ("igg 4") OR ("IgG4") OR ("IgG4 level") OR ("IgG4 Serum") OR ("immunoglobulin 4") OR ("Immunoglobulin G4")) OR AB=(("gamma g4") OR ("igg 4") OR ("IgG4") OR ("IgG4 level") OR ("IgG4 Serum") OR ("immunoglobulin 4") OR ("Immunoglobulin G4")) OR AK=(("gamma g4") OR ("igg 4") OR ("IgG4") OR ("IgG4 level") OR ("IgG4 Serum") OR ("immunoglobulin 4") OR ("Immunoglobulin G4")))
