# Supplementary material for: Association between serum immunoglobulin G4 levels and Graves’ ophthalmopathy: a meta-analysis
Source: Front Endocrinol (Lausanne). 2026 Sep 10;17:1837951. doi: 10.3389/fendo.2026.1837951 (PMC13600816; doi:10.3389/fendo.2026.1837951)
Supplement: Supplementary file 2 [file Supplementaryfile2.docx]

**Forest plot of subgroup analysis of the association between serum IgG4 levels and Graves' eye disease (GO)**

**Study Region**

GO vs GD (1: Asia, 2: Europe, 3: Middle East)

GO vs Normal (1: Asia, 2: Europe, 3: Middle East)

Active GO vs Inactive GO (1: Asia, 2: Europe, 3: Middle East)

High IgG4 vs Lower IgG4 (1: Asia, 2: Europe, 3: Middle East)

**Study Design**

GO VS GD (1: Cross sectional study, 2: Prospective observational study)

GO VS Normal (1: Cross sectional study, 2: Prospective observational study)

High IgG4 vs Lower IgG4 (1: Cross sectional study, 2: Prospective observational study)

**Assay Method**

GO vs GD (1: Turbidimetric assay, 2: ELISA kits Immune enzymatic assay)

GO vs Normal (1: Turbidimetric assay, 2: ELISA kits Immune enzymatic assay)

Active GO vs Inactive GO (1: Turbidimetric assay, 2: ELISA kits Immune enzymatic assay)

High IgG4 vs Lower IgG4 (1: Turbidimetric assay, 2: ELISA kits Immune enzymatic assay)
